# Impact of Physical Exercise on Melanoma Hallmarks: Current Status of Preclinical and Clinical Research

**DOI:** 10.7150/jca.88559

**Published:** 2024-01-01

**Authors:** Claudia Ceci, Celia García-Chico, Maria Grazia Atzori, Pedro Miguel Lacal, Simone Lista, Alejandro Santos-Lozano, Grazia Graziani, José Pinto-Fraga

**Affiliations:** 1 Department of Systems Medicine, University of Rome Tor Vergata, Rome, Italy.; 2 i+HeALTH Strategic Research Group, Department of Health Sciences, Miguel de Cervantes European University (UEMC), 47012 Valladolid, Spain.; 3 Laboratory of Molecular Oncology, IDI-IRCCS, Rome, Italy.

**Keywords:** melanoma, cancer hallmarks, physical activity, immune checkpoints, BRAFi/MEKi

## Abstract

In recent years, accumulating evidence from preclinical and clinical studies consistently indicated that physical activity/exercise plays a crucial role in reducing the incidence and recurrence of various malignancies, by exerting a beneficial modulation of cancer hallmarks. Moreover, physical activity is suggested to attenuate certain adverse effects of anticancer therapy, including the reduction of cardiovascular toxicity and symptoms related to depression and anxiety, among others, while preserving muscular strength.

In the case of melanoma, the relationship with physical activity has been critically debated. Historically, several cohort studies and meta-analyses reported a positive association between physical activity/exercise and melanoma risk. This association was primarily attributed to outdoor activities that may expose the skin to UV radiation, a well-known risk factor for melanocyte transformation. However, more recent evidence does not support such association and recognizes physical activity/exercise role in both melanoma prevention and progression. Nevertheless, sun protection is recommended during outdoor training to minimize UV radiation exposure.

This narrative review summarizes preclinical and clinical data about physical activity effects on melanoma hallmarks. Specifically, experimental evidence is reported concerning (*i*) invasion and metastasis, (*ii*) reprogramming of energy metabolism, (*iii*) angiogenesis, (*iv*) resistance to cell death, (*v*) evasion from immune destruction, and (*vi*) tumor-promoting inflammation.

## Introduction

In the last years, physical activity/exercise was reported to significantly affect cancer prevention and progression, as it lowers both the incidence and recurrence of different tumor types 1-6. Epidemiological studies show evidence of decreased risk of esophagus, stomach, colon, breast, endometrium, bladder, and kidney cancers in association with an active lifestyle, and a possible protective function against ovary, prostate, and pancreatic cancer (World Cancer Research Fund International, available at https://www.wcrf.org) 7. Accordingly, the public health authorities promote regular physical activity/exercise as a component of cancer prevention, due to demonstrated roles in the regulation of body weight and hormone balance, and ability to modulate biological pathways, including but not limited to DNA repair mechanisms, response to oxidative stress or inflammation, and to sustain a proper immune system functionality 8. However, no conclusive data have been reported concerning the adequate intensity and optimal duration of physical activity required to significantly reduce the risk of cancer development or progression. Furthermore, the amount of physical exercise positively affecting the prognosis of cancer seems to strongly differ among tumor types 9.

Melanoma is an aggressive tumor with a marked metastatic potential, originating from cutaneous, uveal, or mucous melanocytes. The incidence of cutaneous melanoma is increasing worldwide (according to the latest statistics, around 325,000 new cases, with a male predominance, and 57,000 deaths have been reported in 2020 10,11). For what concern treatment and prognosis, early-stage melanoma gains benefit from surgical procedures, with five-year survival rate for localized melanoma (stage 0 - II) being of 98.4%. In patients affected by unresectable or late-stage metastatic melanoma, the five-year survival rate significantly improved in the last decade, thanks to treatment based on immune checkpoints inhibitors (ICIs) and serine/threonine-protein kinase BRAF/mitogen-activated protein kinase kinase (MEK) inhibitors (BRAFi/MEKi). The first ones represent the main breakthrough in cancer immunotherapy, the latter (vemurafenib, dabrafenib, and encorafenib as BRAFi; trametinib, cobimetinib, and binimetinib as MEKi) are the standard of care of advanced melanoma carrying *BRAF-V600* activating mutations. BRAF is a serine/threonine protein kinase which stimulates the mitogen-activated protein kinases/extracellular signal-regulated kinase (MAPK/ERK) signaling pathway 12; about 50% of total melanomas harbors activating mutations of *BRAF*, in over 90% of cases being the *V600E* mutation, i.e., the mutation of valine 600 to glutamic acid 13,14. Among ICIs, the human anti-cytotoxic T lymphocyte-associated protein 4 (CTLA-4) monoclonal antibody (mAb) ipilimumab was the first approved by the U.S. Food and Drug Administration (FDA) and the European Medicines Agency (EMA) in 2011 15,16 for the treatment of late-stage melanoma, followed, few months later, by the BRAFi vemurafenib 17,18. In 2014, two other ICIs targeting the programmed cell death 1 (PD-1) protein, nivolumab and pembrolizumab, were approved 19-21 and showed higher efficacy than the anti-CTLA-4. Furthermore, the combination of ipilimumab with nivolumab was associated with even improved outcomes, compared to the single agents. Nowadays, the five-year overall survival rates, reported by the phase 3 randomized clinical trial Checkmate 067, are 26%, 44%, and 52% for ipilumumab, nivolumab, and their combination, respectively 22. Moreover, the latest published results, with a minimum follow-up of 6.5 years, confirmed the higher long-term benefit of the anti-CTLA-4/anti-PD-1 antibody combination 23,24. Concerning BRAF mutated melanoma, the COMBI-d and COMBI-v (dabrafenib plus trametinib), BRIM-7 (vemurafenib/cobimetinib), and COLUMBUS (encorafenib/binimetinib) trials, testing the different BRAFi/MEKi combinations, showed five-year survival rate of 34-39% 25-27.

Physical activity was first associated to a higher risk of malignant melanoma development, since training in outdoor settings may enhance the exposure to the main risk factor for melanocytes transformation, such as ultraviolet (UV) radiation 28-31. More recently, in line with data coming from other tumor types, physical activity has been acknowledged to be an important factor in preventing malignant melanoma onset and progression. Moreover, no direct positive association between physical activity, melanoma risk, and thickness at diagnosis (depth of melanoma from the surface of the skin), was reported in a population-based analysis, from a prospective cohort study carried out on the population-based Norwegian women and Cancer (NOWAC) cohort aged 30-75 years 32,33.

Our research group recently summarized the current preclinical and clinical evidence concerning the effects of exercise training on breast cancer hallmarks 34.

Aim of the present narrative review is to summarize the currently available literature defining how physical activity might affect melanoma onset, progression, and recurrence, based on the modification of certain cancer hallmarks by regular training (details about the systematic examination of existing literature on the topic are reported in **Figure S1**).

To our knowledge, no published literature reviews attempted, up to now, to examine the connection between the dynamically evolving definition of cancer hallmarks 35 and the characterization of physical exercise impact on a common, and often aggressive, type of cancer, such as melanoma. Therefore, by focusing on the exercise-induced modification of biological biomarkers, this report may help in understanding the potential underlying molecular mechanisms through which physical activity may affect melanoma biology. In the final part of this review, we sought to critically explore whether a non-sedentary lifestyle might enhance the anti-melanoma potential of currently accessible or under investigation pharmacological strategies.

## Melanoma hallmarks modulated by physical activity

### Invasiveness/metastasis

**Figure 1:**
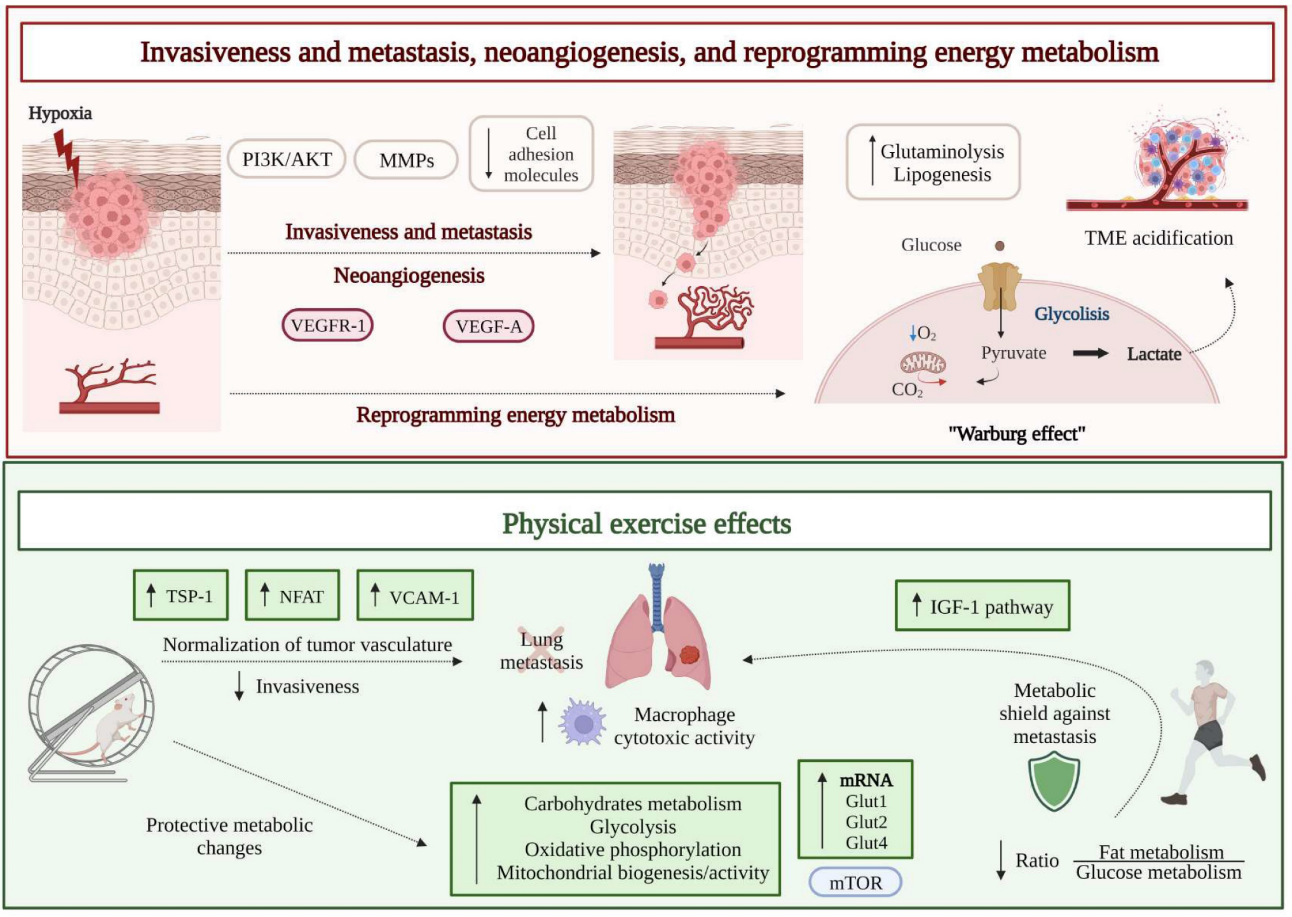
Schematic drawing of melanoma hallmarks critically depending on the hypoxic state that accompanies the growth of the tumor mass, i.e., invasion and metastasis, neoangiogenesis, and reprogramming energy metabolism (upper part): the increase in tumor mass causes a decrease of oxygen availability and upregulation of proangiogenic (VEGF-A) and prometastatic (MMPs) factors, under the stimulation, among others, of the PI3K/AKT pathway. This response, leading to pathological angiogenesis and metastatic spreading, is crucial for the tumor to overcome hypoxia. However, the new blood vessels show a chaotic and tortuous structure. Moreover, cancer cells modify their metabolism and use glycolysis even under aerobic conditions (“*Warburg effect*”), with consequent increased production of lactate and acidification of the tumor microenvironment. Effects of physical activity on melanoma invasion and metastasis, neoangiogenesis, and reprogramming energy metabolism (lower part): an altered expression of soluble factors responsible for vascular remodeling, i.e., NFAT and TSP-1, was observed by means of *in vitro* experimental conditions that mimic the increase in blood flow observed *in vivo* in association with physical exercise. Moreover, upregulation of VCAM-1 in endothelial cells and a significant vessel normalization were observed *in vivo* in the tumor-associated vasculature of melanoma murine models performing treadmill exercise, compared to sedentary control animals. The consequent reduced invasiveness limits metastatic spreading. Melanoma metastasis also seems to be hampered by mTOR mediated protective metabolic changes induced by physical activity, like an increase in glucose metabolism, as evidenced both in animals and in humans. Abbreviations. Glut: glucose transporter; IGF-1: insulin-like growth factor-1; NFAT: nuclear factor of activated T cells; MMPs: matrix metalloproteinases; TME: tumor microenvironment; TSP-1: thrombospondin-1; VCAM-1: vascular cell adhesion molecule-1; VEGF-A: vascular endothelial growth factor A; VEGFR-1: vascular endothelial growth factor receptor 1.

Melanoma often spreads to nearby lymph nodes and/or distant sites, leading to the formation of metastases in lungs, liver, brain, and bones. Despite the recent improvement of clinical outcomes obtained with immunotherapy or BRAFi/MEKi, advanced stage melanoma is still associated with a poor prognosis in a remarkable percentage of patients 36. Hypoxia, in addition to promote malignant transformation of melanocytes, critically supports metastasis formation (**Figure 1**). Mechanistically, the major events contributing to melanoma cell motility from primary site include increased expression of matrix metalloproteinases (MMPs) and loss of adhesion molecules, like integrins and cadherins, which physiologically mediate cell attachment to the basement membrane and cell-cell interactions. In detail, E-cadherins are progressively reduced, followed by the concomitant upregulation of N-cadherins, supporting melanoma cells migration, under the control of the phosphoinositide-3-kinase/AKT (PI3K/AKT) pathway 37.

The results of several preclinical studies indicate that physical exercise can primarily modulate tumor hypoxia and decrease tumor growth and metastasis 38 (**Figure 1**). A first preclinical study investigated the effect of physical exercise on melanoma colonization of the lungs by using male C57Bl/6 mice injected intravenously with syngeneic B16 melanoma cells and assigned to the following groups before tumor challenge: Ex-30 (running 30 minutes), Ex-F (running and fatigued), Ex-F-24 h (running and fatigued for 24 hours before tumor initiation), or Control (resting control above the treadmill). In all groups, lungs were removed 7-10 days after tumor cell injection, and count of tumor foci revealed that Ex-F and Ex-F-24 h cohorts had fewer tumors than either Ex-30 or Control groups. Moreover, alveolar macrophages, removed from sacrificed mice and co-cultured *in vitro* with B16 cells, were more able to reduce melanoma proliferation when obtained from mice of the Ex-F group, compared to animals belonging to the Control group, suggesting that exercise not only protects against lung tumor metastases, but also enhances the cytotoxic activity of alveolar macrophages 39. Years later, the same group used a modified exercise protocol 40, including six consecutive days of running on treadmill (1 hour/day) to better evaluate the impact of short-term, moderate training on the establishment of melanoma lung metastases, in combination with the consumption of oat β-glucan (a dietary fiber found in the cell wall of fungi and yeast, endowed with some anticancer effects 41). Although without additive effects, both short-term moderate intensity exercise and intake of oat β-glucan decreased the *in vivo* metastatic spread of B16 melanoma, at least partly due to an increased macrophage antitumor cytotoxicity 40.

Opposite results came from a study based on voluntary training on a non-motorized wheel in an experimental metastasis model represented by intravenously injected B16BL/6 melanoma cells in male C57BL/6 mice. When frequency, duration, and intensity of the exercise were determined by the animals themselves, despite variation in the daily physical activity that could affect the outcomes of the experiment, it was possible to eliminate the stress associated with a forced running by treadmill. However, in this experimental setting, no differences were identified in terms of number and size of lung metastases between sedentary and running mice 42.

A more recent study reported that physical exercise can induce internal organs to reprogram their metabolic activity and increase their nutrient demand, thus protecting them from metastasis, through a reduction of nutrient availability 43. In detail, three preclinical models of melanoma were established, by performing subdermal, intracarotid, or intrasplenic Ret-melanoma cell injection in C57BL/6JRccHsd mice, after an exercise training protocol on the treadmill. In this study, female animals were used based on their enhanced metabolic response to exercise. In all the experimental models, exercise before cancer cell injection protected animals against metastasis to distant organs. In the same article, a parallel prospective human study on 2734 cancer-free participants, with a follow-up of 20 years, allowed to prove that high-intensity exercise significantly reduced the incidence of invasive cancers by 73%, compared to inactivity, supporting the inverse correlation between training and development of invasive malignancies 43.

### Reprogramming of energy metabolism

In order to survive in situations of fluctuating oxygen levels, cancer cells modify their metabolism and use glycolysis even under aerobic conditions, a process known as “*Warburg effect*” associated with consequent production of high lactate levels and acidification of the tumor microenvironment 44. Enhanced glutaminolysis and changes in lipid metabolism (i.e., increased lipogenesis and fatty acid absorption) further contribute to metabolically affect tumor cell growth, spread, and resistance to chemotherapy 45 (**Figure 1**).

Exercise training is able to target the specific metabolism of tumor cells, namely the Warburg-type high glycolytic metabolism, in a mode-, intensity-, duration-, and frequency-dependent manner. High-intensity anaerobic exercise carefully performed according to individual specifications, can achieve better results than moderate-intensity aerobic exercise in inhibiting glycolysis and reducing tumor growth 46. Because of the atypical metabolic profile of tumors, cancer and healthy cells also differ in their response to caloric restriction. Cancer cells are unable to adapt to the lack of nutrients and maintain a sustained proliferation even in case of fasting, while normal cells switch to a maintenance program conferring resistance to stress 47. Indeed, fasting for 72 h protected melanoma- and glioma-bearing mice against the toxicity of high-dose chemotherapy 48. On the other hand, by reducing available glucose, energy restriction may affect cancer cells relying on glycolysis through the “*Warburg effect*” more than quiescent normal cells. Therefore, reduced food intake could enhance response to chemotherapy, alone or in combination with immunotherapy, and preclinical data support the adjuvant use of caloric restriction. Of note, caloric restriction promotes both life expectancy and healthspan, whereas burning of calories through exercise only prologs healthspan 49, suggesting molecular effects of caloric restriction beyond energy balance.

The aforementioned study 43, which examined the role of physical exercise in protecting internal organs from colonization by metastatic melanoma cells, postulated that the reprogramming of host organs metabolic habits hampers colonization by cancer cells (**Figure 1**). In order to verify such hypothesis, a proteomic analysis of internal organs (lungs, lymph nodes, liver, and muscle) was performed in mice exposed to forced training on treadmill prior to melanoma initiation. As reported, all trained animals showed exercise-induced metabolic alterations, in terms of upregulation of carbohydrates metabolism, glycolysis, oxidative phosphorylation, and mitochondrial biogenesis/activity, in all the explored organs. Moreover, the expression levels of glucose transporters (GLUTs) mRNAs (*Glut1*, *Glut2*, and *Glut4*), required for glucose uptake by cells, were significantly increased in the organs from active mice, compared to inactive mice 43. Such protective metabolic changes induced by physical activity required the participation of the mTOR pathway, since its inhibition reversed the metabolic shield. Indeed, when primary cells from lungs of active mice were treated with the mTOR inhibitor rapamycin and co-cultured with B16 melanoma cells, the metabolic advantage was lost, and tumor growth was resumed.

A similar proteomic analysis of plasma samples obtained after 30 minutes treadmill high-intensity training from a small cohort of healthy 43, routinely active human volunteers (25-45 years of age, performing weekly endurance exercise), revealed a significant upregulation of proteins belonging to the insulin-like growth factor-1 (IGF-1) pathway. In another small cohort, the high-intensity exercise affected the ratio between fat and glucose utilization, with the latter rising as the intensity of exercise increased 43. Thus, data from both cohorts suggested an increased consumption of carbohydrates during high-intensity exercise. The same study followed an initially cancer-free prospective cohort of 2734 participants for 20 years and recorded self-reported descriptions of exercise intensity and duration. Epidemiological analysis showed that the risk of metastatic cancer was significantly reduced by high-intensity exercise (by up to 73% compared with inactivity). Therefore, the shift in macronutrient utilization observed by proteomic analysis in small cohorts performing regular exercise was hypothesized to be a protective factor against the development of highly metastatic tumors in humans 43.

### Neoangiogenesis

The transition from a proliferative to an invasive behavior, which is part of malignant melanoma progression, critically relies on the formation and growth of new blood vessels, i.e., neoangiogenesis. Indeed, the newly formed tumor-associated vasculature provides an excellent route by which cancer cells can spread and generate metastases in distant organs from the primary tumor. The increase in tumor mass is paralleled by the decrease of oxygen availability, to which the tumor itself and the tumor microenvironment respond by producing an excess of proangiogenic factors. Neovessel formation is typically stimulated by an excessive release of the vascular endothelial growth factor-A (VEGF-A) from hypoxic tumor cells. Upregulation of VEGF-A/vascular endothelial growth factor receptor 1 (VEGFR-1) signaling pathway is crucial for pathological angiogenesis and metastatic spreading 50,51 (**Figure 1**). The new vascular system, however, lacks a regular and hierarchical organization, exhibiting a chaotic and tortuous structure 52,53. Such altered circulatory system paradoxically perpetuates tumor hypoxia and contributes to the acquisition of an even more aggressive tumor phenotype, prone to metastasis and resistance to anticancer treatments, since it hampers a proper drug distribution to the tumor site. Accordingly, therapeutic approaches have been developed in the past years, combining chemotherapy with antiangiogenic agents, aimed at normalizing tumor vasculature and improving drug delivery 54,55.

Considering that, together with the chemical signal provided by VEGF-A, mechanical signals generated by an increased blood flow and pressure contribute to the development of the tumor vasculature, the acquisition of a proper vessel functionality has been assumed to be potentially favored by modifying these parameters. In turn, aerobic physical exercise has been considered as a strategy that may support the increase in blood flow. In a recent study, B16F10 melanoma from post-tumor implantation exercised C57BL/6 mice (aerobic moderate intensity treadmill running) showed a significant increase in both vessel length and blood supply 56; consequently, the reduction in tumor growth induced by a chemotherapeutic agent (doxorubicin) in exercised mice was significantly higher, compared to animals treated with chemotherapy alone. By using *in vitro* experimental conditions that mimic a stress in endothelial cells like that resulting from an increased blood flow *in vivo*, an altered expression of soluble factors responsible for vascular remodeling was observed. In particular, activation of nuclear factor of activated T cells (NFAT) transcription factor and augmented secretion of thrombospondin-1 (TSP-1), a key antiangiogenic protein acting as a critical player in tumor vascular normalization induced by exercise, were reported 56 (**Figure 1**). However, post-implantation exercise alone in B16F10 melanoma bearing mice did not affect vascularity, perfusion, hypoxia, and tumor growth rate. Indeed, the hypoxic areas, number of perfused vessels, and CD31-positive vessel density were unchanged in tumors from exercising mice on a running wheel, compared to non-exercising mice 57. Therefore, the influence of exercise alone on melanoma angiogenesis and perfusion appears to be minor.

More recently, normalization of tumor vasculature by aerobic exercise gained novel attention, and a possible involved mechanism was identified, such as the upregulation of the expression of vascular cell adhesion molecule 1 (VCAM-1) by endothelial cells in B16F10 and YUMMER 1.7 murine models of melanoma 58. In an experiment involving male C57BL/6 mice, a regimen of treadmill exercise (i.e., treadmill running for 45 minutes/day for 12-14 days post-tumor implant) resulted in a significant remodeling of melanoma-associated vasculature. Such a remodeling was characterized by a reduction in vessel permeability and no change in tumor perfusion, compared to sedentary control animals 58.

### Resistance to cell death

**Figure 2:**
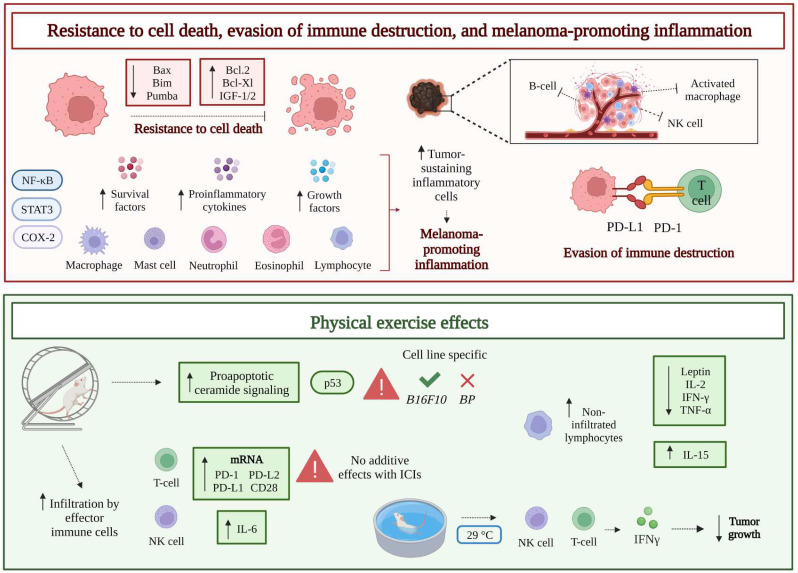
Schematic drawing of other melanoma hallmarks: resistance to cell death, evasion of immune destruction (due to blockade of anticancer immunity), and melanoma promoting inflammation (supporting tumor-sustaining pro-inflammatory cells) (upper part). Typically, cancer cells show defects in the apoptotic machinery, i.e., up-regulation of anti-apoptotic factors, like Bcl-2 or Bcl-Xl, and down-regulation of pro-apoptotic factors, like Bax or Bim. Moreover, the anticancer response mounted by the immune system is hampered, due to an increased activation of immune checkpoints (PD-L1/PD-1 axis). Finally, the promotion of inflammatory response, with consequent excessive production of cytokines and growth/survival factors, further contributes to support melanoma evolution. The transcription factors NF-κB and STAT3, and the enzyme COX-2 are key molecular players in the enrollment of pro-inflammatory cells by the tumor. Effects of physical activity on resistance to cell death, evasion of immune destruction, and melanoma promoting inflammation: by upregulating the canonical p53 pathway, physical activity has been reported to increase the pro-apoptotic signaling of ceramide in a melanoma murine model. Infiltration of the tumor by effector immune cells, like T, NK, and dendritic cells, and the increased proliferation of tumor non-infiltrated lymphocytes represent additional effects of physical activity, together with the decrease of serum levels of leptin (satiety hormone associated with pro-inflammatory, anti-apoptotic, and pro-angiogenic actions) and pro-inflammatory cytokines (IFN-γ, IL-2, and TNF-α). On the other hand, acute exercise may enhance IL-15 levels. Finally, in preclinical studies physical activity seemed to directly upregulate the expression of immune checkpoints (PD-1, PD-L1, and PD-L2) but did not synergize with PD-L1 or PD-1 inhibitors. In the context of aquatic exercise, the amount of splenocytes from mice exercised in thermoneutral water (TT, 29°C) was significantly increased, compared to animals exercised in body temperature water (BT, 36°C), particularly involving CD8+ T and NK cells, with consequent enhanced release of IFNγ. Abbreviations. COX-2: ciclooxygenase-2; IFN-γ: interferon γ; IGF-1/2: insulin-like growth factor-1/2; IL-2: interleukin-2; IL-6: interleukin-6; IL-15: interleukin-15; NK: natural killer; PD-1: programmed cell death protein 1; PD-L1: programmed death-ligand 1; TNF-α: tumor necrosis factor α.

The uncontrolled cell proliferation associated with malignant transformation is often correlated with acquired resistance to apoptotic cell death, through several molecular mechanisms, including the loss of p53 tumor suppressor activity, upregulation of anti-apoptotic (Bcl-2, Bcl-Xl) or pro-survival (IGF-1/2) factors, downregulation of pro-apoptotic factors (Bax, Bim, Puma), and inactivation of effector caspases (**Figure 2**).

Among tumor cell intrinsic death mechanisms, ceramide-promoted cell death has been identified. Ceramide is a bioactive lipid that regulates cell death/survival, and human melanoma seems to exhibit a reduced expression of ceramide synthase 6 (CerS6), the enzyme which generates the pro-apoptotic form of the molecule (C16-ceramide) 59. In a recent study, a two-week moderate aerobic treadmill-based exercise was demonstrated to increase the pro-apoptotic ceramide signaling pathway and sensitivity to doxorubicin in B16F10 melanoma tumor-bearing male C57BL/6J mice, by upregulating canonical p53 signaling and DNA fragmentation. However, promotion of the pro-apoptotic ceramide pathway was not observed in a second melanoma murine model (BP murine melanoma), thus suggesting that the effect of physical exercise on cancer cell death can vary, depending on the melanoma preclinical model tested 60 (**Figure 2**).

### Evasion of immune destruction

Melanoma is a highly immunogenic tumor, whose development involves sequentially connected phases 61: *i*) *elimination* of tumor cells by natural killer (NK), dendritic, T, and B cells mediated immunosurveillance; *ii*) establishment of an *equilibrium* between tumor and immune cells; *iii*) final *escape*, or immune evasion, due to exhaustion of the tumor immunosurveillance, that would mainly depend on reduction of tumor-associated antigen expression and increase of inhibitory molecules production (e.g., programmed death-ligand 1 [PD-L1]) by the tumor, or increased expression of cognate checkpoint receptors (PD- 1) by immune cells 62,63 (**Figure 2**). Accordingly, the recent therapeutic approaches, including CTLA-4 and PD-1 inhibitors (i.e., ICIs), aimed at reinforcing the molecular interaction between effector immune cells and tumor cells, have dramatically improved the outcome of metastatic melanoma patients 22-24. Nevertheless, the recruitment of tumor antigen-specific T cells to the tumor site is crucial for a significant response to ICIs. Since chemokines play a major role in recruiting tumor infiltrating lymphocytes 64, melanomas showing low expression levels of chemokine receptors (CXCR3 and CCR5) ligands are poorly infiltrated by T cells 65. Moreover, other complications may hinder the chemotaxis of anticancer T cells, among which the previously mentioned abnormal tumor vasculature and the consequent poor tumor perfusion.

According to the accumulating data about exercise effects on cancer, a potential role of physical activity in enhancing the outcomes of anticancer immune response and immunotherapy has been explored 66. A recent comprehensive analysis suggested that regular exercise may increase immune function, particularly T cell activity, thereby potentially reducing the incidence of cancer among physically active people 67.

Preclinically, voluntary wheel running has been reported to reduce tumor growth in a B16 melanoma model established in female C57BL/6 mice, by significantly increasing infiltration of the tumor by effector immune cells, including T, NK, and dendritic cells. Specifically, the release of epinephrine during exercise mobilized immune cells into the circulation and promoted NK cell tumor infiltration that inversely correlated with tumor burden 68. In athymic mice lacking efficient T cells but retaining NK cells, the reduction in B16 tumor volume by 6-weeks long training was maintained, even though to a lesser extent than in wild-type immune-competent exercised mice, indicating that T cells control tumor growth, but are dispensable in the context of exercise-promoted reduction of tumor burden. In contrast, depletion of NK cells completely nullified the inhibitory impact of running on tumor growth 68. Such evidence is in line with the general idea that, among immune cells, NK cells are the most responsive to exercise. Notably, a substantial mobilization of NK cells into the bloodstream occurs within minutes from the beginning of training, and this phenomenon may potentially contribute to the exercise-mediated protection against cancer 69.

Moreover, increased expression levels of mRNAs coding for ligands (H60a, Clr-b, MULT1), cytokines (IFNγ, IL-2, IL-15), and chemokines (CXCL10, CCL3, CX3CL1) involved in NK cell activation and chemotaxis, were reported in tumors from exercised mice. IL-6 plasma concentrations were also found to markedly increase during exercise, being recognized as an additional factor involved in tumor homing: blockade of training-induced IL- 6 by specific antibodies reduced the inhibitory effect on tumor growth and the infiltration of tumors by NK cells 68. In another preclinical study, CD8+ T cells from non-exercising or exercising mice were transferred to sedentary female C57BL/6J mice that had been inoculated with ovalbumin (OVA) expressing melanoma cells (B16F10-OVA) 70. The recipient animals receiving T cells from exercising donors showed increased survival and decreased tumor growth rate, compared to the sedentary melanoma carrying animals that received T cells from non-exercising mice.

Results obtained from studies emphasizing the exercise-induced mobilization and activation of cytotoxic immune cells as a crucial factor contributing to the exercise-dependent regulation of tumor growth in melanoma *in vivo* models 57,68,71 have been further reinforced by a recent study addressing the transcriptome of B16F10 tumors in female C57BL/6 mice, under both standard chow and high-fat dietary conditions 72. As expected, markers of macrophages (CD68, CD74, and CD209), NK (NKG2D and NK1.1), and T cells (CD8, PDCD10, perforin, GrmM) were upregulated by voluntary wheel running in chow-fed mice. However, the exercise-amplified expression of markers related to myeloid and NK cells was attenuated in high-fat diet mice, while the induction of T cell markers exhibited, comparatively, a lower degree of alteration. Therefore, in line with epidemiological results that underscore the potential of exercise to diminish cancer risk and severity 30, voluntary wheel running exerted a restraining effect on tumor growth irrespective of dietary conditions; such an effect was also largely independent of body mass index. However, exercise-induced innate immune recognition of tumors was reduced in association with high-fat feeding 72.

Based on a more recent study, aerobic exercise differentially regulated antitumor immune response and melanoma growth since it significantly increased CD8+ T cell infiltration and reduced tumor size in YUMMER but not in B16F10 murine melanoma models 58.

In humans, exercise has a substantial impact on the immune system functionality: long periods of intensive training seem to depress immunity, whereas moderate intensity physical activity appears to be beneficial, in terms of transient increase of leukocyte count 73. Leukocytosis in response to exercise was first reported about two decades ago 74. More recently, it was shown that leukocyte subsets are differentially mobilized into the peripheral blood, in a manner dependent upon exercise intensity and duration 75. Albeit initially not believed to hold clinical relevance, leukocytosis induced by exercise, in particular the “immuno-enhancing” effect of single bouts of moderate intensity exercise, has been recognized to potentially improve health outcomes in elderly, obese, and patients with chronic viral infections, as well as to represent a tool to boost the immune system in cancer patients receiving immunotherapy 73. Furthermore, the previously discussed increase in blood flow, due to normalization of the tumor blood vessels, observed in conjunction with physical activity, can further increase the infiltration of immune cells within the tumor mass.

Voluntary physical activity, in addition to decrease melanoma growth in B16 tumor-bearing female C57BL/6NTac mice, also seemed to directly upregulate the expression of immune checkpoints. In detail, the mRNA expression levels of PD-1 and cognate ligands, PD-L1 and PD-L2, were augmented by 2.9-, 2.3-, and 3.1-fold, respectively, whereas mRNAs codifying for the CD28 co-stimulatory molecule and its ligands (B7.1 and B7.2) showed an increase of 2.2-, 2.8-, and 2.9-fold, respectively 71.

Although also patients with PD-L1 negative tumor may benefit from the treatment with ICIs, high PD-L1 expression in the tumor is assumed as a promising predictive biomarker of response in several solid cancers, including cutaneous melanoma 76-78. Therefore, on this basis, exercise-induced up-regulation of PD-L1 is expected to potentially improve melanoma response to anti-PD-1/PD-L1 therapies. However, the combination of physical activity with anti-PD-L1 or anti-PD-1 approaches did not induce further inhibition of tumor growth. B16 melanoma carrying female C57BL/6NTac mice received 100 mg of a PD-L1 inhibitor or PBS, thrice a week following tumor challenge, after randomization to cages with or without running wheels. During the 5 weeks preceding tumor injection, animals ran 0.9-5.8 km daily, without significant difference in running wheels utilization between the PD-L1 inhibitor and PBS groups. Tumor growth was significantly suppressed by wheel running (by 72%, p = 0.13), although without statistically significant interaction with the anti-PD-L1 therapy (83% tumor growth reduction with the combinatorial approach, p < 0.05). The same was observed following wheel running combined with the anti-PD-1 treatment, when running and control mice carrying B16 melanoma tumors were treated with 200 μg anti-PD-1 or PBS, twice a week post- tumor implantation. In this case, animals ran 4-8 km per day, and the anti-PD-1 group ran significantly more than the PBS group. Despite a significant reduction of tumor growth induced by physical activity (40%, compared to the inactive group receiving PBS, p < 0.05), also in this case the combination of training with the anti-PD-1 did not result in additive effects (50% tumor growth inhibition, exercising *versus* sedentary groups, p = 0.07) 71. In agreement with these findings, in YUMMER and B16F10 melanoma models aerobic exercise did not enhance the antitumor activity of an anti-PD1 treatment 58.

While not improving the efficacy of immunotherapy against melanoma, the exercise and anti-PD-1 combinatorial approach induced modifications of the tumor immune microenvironment, which differed according to the tumor model tested 79. Indeed, post-implant exercise alone (on running wheels associated with a digital count of revolutions) was found to significantly decrease the number of tumor resident CD8+ T cells in a breast cancer *in vivo* model (EO771 cells in female C57BL/6 mice), but not in the case of melanoma (B16F10 cells in female C57BL/6 mice). Moreover, exercise reduced the amount of CD8+ T cells within the total T CD3+ cell population in both models, indicating a general shift towards a more immunosuppressive phenotype. On the contrary, in combination with an anti-PD-1 agent, exercise increased the fraction of CD8+ T cells in EO771 but not in B16F10 melanoma. No changes were observed in the amount of tumor infiltrating NK cells between non-exercising and exercising mice in both tumor types, and regardless of the anti-PD1 treatment 79. These data are in contrast with the study by Pedersen et al., in which exercise increased the number of intratumoral NK cells in B16F10 melanoma 68. As suggested by the authors, a possible explanation for such discrepancy has to be found in the time of exercising, being pre-implantation, in one case, and post-implantation, in the other case 79; therefore, it seems that an exercise-based pre-conditioning, before tumor cells injection, may be required to promote NK cell infiltration.

A particular area of research is focused on the beneficial effects of aquatic exercise on anticancer immunity. In this context, the effect of water temperature on the immune response triggered by melanoma has been studied in male C57BL/6 tumor-bearing mice swimming in thermoneutral (TT, 29°C) or body (BT, 36°C) temperature for 3 weeks, 6 days a week, for 30 minutes 80. Interestingly, tumor growth was significantly impaired in mice that swam in TT as compared to mice that exercised in BT water or control mice. When testing the number of immune cells involved in the antitumor immune response, the total amount of splenocytes significantly increased in the group exercised in TT water. Moreover, FACS analysis showed that such significant increase involved CD8+ T cells, γδT cells (T cell subpopulation with immune surveillance activity), natural killer T cells (NKT), and NK cells (1.6, 2.5, 2.5, and 2-fold, respectively). Furthermore, CD8+ T cells from the TT group were associated with a significantly enhanced release of IFNγ, compared to CD8+ T cells from the BT group (**Figure 2**). Finally, soluble γc protein (sγc), known to inhibit antitumor immune response 81, showed a relatively late down-regulation in the serum of resting mice, compared to the active group, and a significantly higher rate of decline in mice exercised in TT water than in animals exercised in BT water 80.

### Melanoma-promoting inflammation

While the inflammatory response typically serves as a defense mechanism triggered in response to trauma and/or invasion by infectious agents, and usually subsides with tissue repair, chronic inflammation has the potential to foster carcinogenesis, which might be attributed - at least partly - to an excessive production of cytokines and growth/survival factors. Additionally, sustained chronic inflammation can impair proper mitochondrial function that plays a critical role in the regulation of energy-generating pathways and apoptosis. In turn, the compromised “mitochondrial fitness” may contribute to cancer initiation, while an active lifestyle may enhance the mitochondrial function and reduce the risk of cancer 82.

An inflammatory microenvironment is crucial in both cancer onset and evolution. Tumor-associated monocytes/macrophages, activated T lymphocytes, mast cells, neutrophils, and eosinophils are the critical inflammatory cells sustaining malignancies, through the release of growth factors, extracellular proteases, chemokines, proangiogenic factors 83. In turn, NF-κB and STAT3 transcription factors, together with the pro-inflammatory isoform of the cyclooxygenase (COX-2) enzyme, are key molecular players in the enrollment of pro-inflammatory cells by the tumor and production of pro-inflammatory mediators 84,85 (**Figure 2**).

Since obesity can induce a chronic and systemic inflammatory status that promotes cancer, the effect of moderate physical exercise on a treadmill has been tested, in terms of impact on tumor non-infiltrating lymphocyte's function and tumor growth, by using a murine model (female C57BL/6 mice) of melanoma maintained on a high-fat diet. As expected, the high-fat diet not only led to obesity, but also promoted melanoma growth (twice increase, compared to mice with a balanced diet), while a concomitant moderate physical exercise significantly reduced tumor growth 86. Moreover, exercise increased the proliferation of tumor non-infiltrated lymphocytes, regardless of the dietary regimen, and decreased the serum levels of leptin, in both tumor-bearing and tumor-free high-fat diet-fed animals. Leptin is a satiety hormone associated with pro-inflammatory, anti-apoptotic, and pro-angiogenic actions, thus exerting a relevant role in the promotion of tumor growth 87; indeed, its serum concentrations were higher in mice fed with a high-fat diet. Finally, a significantly reduced secretion of pro-inflammatory cytokines (IFN-γ, IL-2, and TNF-α), involved in the Th1 response and recruitment of other leukocytes, was observed in melanoma bearing mice receiving a high-fat diet and on continuous exercise. Therefore, physical activity contributed to decrease the chronic inflammation accompanying both obesity and tumorigenesis. On the other hand, no significant difference was found in IL-4, IL-6, IL-10, IL-17 secretion between control or tumor bearing mice, receiving either the balanced or the high-fat diet, sedentary or exercised 86.

In another study, IL-15 was evaluated across different tumor types as a potential molecular biomarker for prediction of patient prognosis and for evaluating the anticancer effects of exercise 88, by detecting its expression through the Cancer Genome Atlas (TCGA), the Human protein Atlas (HPA), and the Genotype Tissue-Expression (GTEX) databases. At first, IL-15 mRNA expression and protein levels were found to be significantly down-modulated in 12 different tumors, including skin melanoma, compared with normal tissues. On the other hand, a high expression of IL-15 was associated with the prediction of a positive survival outcome in different cancer patients, including those with skin melanoma (**Figure 2**). In line with this evidence, some studies are testing recombinant human IL-15 in cancer patients, including those with metastatic melanoma 89,90. Thus, the observation made in healthy individuals of enhanced IL-15 levels for 10-120 min after acute exercise 91, allowed to establish that the better prognosis reported in regularly training cancer patients may originate from up-regulation of IL-15 expression 88.

Inflammation-related molecules were also suggested as potential prognostic biomarkers by a recent comprehensive bioinformatics analysis that exploited different datasets to identify genes induced by exercise associated with malignant melanoma 92. In particular, the GSE62628 database, released in July 2016 and containing the gene expression profile of melanoma tissues from exercised and unexercised mice, was used to obtain information on exercise-induced genes. By analyzing the whole genome changes in gene expression occurring in malignant melanoma, both before and after voluntary exercise, a total of 1627 differentially expressed genes (DEGs) were identified, with 1285 and 342 genes showing increased and decreased expression, respectively. Analysis of these DEGs, performed through Gene Ontology, demonstrated that they were mainly involved, among other immune/inflammatory processes, in NF-κB, cytokines, or chemokines signaling pathways, and NK cell mediated cytotoxicity 92.

### Physical activity and other melanoma hallmarks

Other hallmarks, not discussed above, have been described in the field of cancer, namely: sustaining proliferative signaling, evading growth suppressors, enabling replicative immortality, genome instability and mutation, unlocking phenotypic plasticity, nonmutational epigenetic reprogramming, polymorphic microbiomes, and senescent cells. However, relevant evidence supporting physical exercise impact on such hallmarks in the context of melanoma is currently scarce or missing.

- *Sustaining proliferative signaling.* Exercise is known to activate MAPK/ERK and PI3K/AKT pathways 93, both involved in the pathogenesis and progression of melanoma 94-96. Specifically, molecular changes that occur during the progression of melanoma also include hyperactivation of PI3K/AKT and inactivation of p53 97. Likewise, high IGF-1 levels activate the PI3K/AKT signaling and decrease apoptosis, which would increase tumor progression 98. In this context, it has been proposed that physical exercise may increase p53 levels and attenuate serum IGF-1 levels 99,100. However, despite the activation of MAPK/ERK and PI3K/AKT pathways, and the lack of data supporting the downregulation of IGF-1 (actually, increased IGF-1 levels were reported in exercising healthy volunteers 43), physical exercise does not seem to promote the proliferative signaling hallmark in melanoma.
- *Evading growth suppressors*. Under physiological conditions, when DNA is damaged, p53, as a “guardian of the genome”, is released from its negative regulatory protein MDM2 through a phosphorylation process. Subsequently, p53 activates genes, such as p21, IGFBP-3, and PTEN. Altogether, p53 and downstream proteins orchestrate a variety of cell responses (DNA repair, cell cycle arrest, cellular senescence, cell death) which, in case of cancer, drive inhibitory molecular processes on its growth 101. In the context of physical activity and modulation of melanoma hallmarks, the previously reported role of p53 in ceramide pro-apoptotic signaling 59 may represent a first indication, to be further investigated, for what concern exercise effect on melanoma evasion of growth suppressors.
- *Enabling replicative immortality*. It is known that exercise attenuates telomere attrition and helps maintaining a balance with oxidative stress and inflammation 102. The maintenance of a healthy body composition has been described as one of the potential mechanisms by which exercise could lead to an attenuated telomere attrition 103. However, to our knowledge, no clinical or preclinical studies have addressed the effects of physical exercise on telomere length or telomerase activity in melanoma.
- *Genome instability and mutations.* Although there is no evidence on the benefits of exercise on this melanoma hallmark, it is known that an excessive telomere shortening not only increases the risk of cancer but also induces genomic instability, by mediating interchromosomal fusion 104. Therefore, the previously mentioned hypothesis, which indicates that exercise, thanks to the maintenance of a healthy body composition, could reduce carcinogenesis by hampering the replicative immortality, could also be applied to this hallmark, but much more information is required.
- *Unlocking phenotypic plasticity*. As repeatedly stated, hypoxia and inadequate blood supply encourage the development of an aggressive cancer phenotype, thus contributing to the failure of systemic treatments 105. It has been hypothesized that temporary systemic acidity, induced by exercise, affects the tumor microenvironment composition *in vivo* and delays the tumor's adaptation to localized hypoxia and acidosis 106. For instance, it is known that M1 macrophages enhance the inflammatory state, while M2 macrophages inhibit it, and in obese male C57BL/6 mice, aerobic physical exercise has been shown to increase the expression of CD163, a specific marker of M2 phenotype 107. However, a recent study in murine melanoma models showed that aerobic exercise caused a phenotypic shift away from the M2 type in the tumor-associated macrophage population 58. Melanoma cells themselves show a high phenotypic plasticity, being able to switch back and forth between various subtypes, including the undifferentiated, neural crest, transitory, and melanocytic subtype 108. However, studies addressing the effect of exercise on this melanoma hallmark are presently limited.
- *Nonmutational epigenetic reprogramming.* Cancer is associated with hypermethylation, and consequent silencing, of tumor-suppressor genes. Exercise may affect cancer development through epigenetic alterations, including DNA methylation 109. Specifically, physical activity has the ability to preserve and/or recover “positive” epigenetic markers that are altered in the case of cancer 110. However, no clinical or preclinical studies have been found in this regard for melanoma. Future research should assess hypo- and hypermethylated genes in melanoma 111 and address the effects of exercise on nonmutational epigenetic reprogramming.
- *Polymorphic microbiomes.* Preclinical studies investigated the potential impact of the intestinal microbiota composition on the pathogenesis of melanoma and its response to immunotherapy 112-114. Increasing research is even focusing on the skin microbiome and its effect on the progression of melanoma 112. In the case of other cancers, such as breast 115 and colorectal cancer 116, exercise was documented to promote antitumor characteristics of the gut microbiome. In the case of melanoma, although there is an ongoing study aiming at examining such effects (available at https://clinicaltrials.gov/ct2/show/study/NCT04866810), results are still unknown.
- *Senescent cells.* Cellular senescence plays an essential role in the relationship between skin ageing and development of melanoma. According to a clinical study involving endurance runners, the increase of senescent cells in cancer-prone organs, observed along with aging, may be prevented by prolonged high-volume, high-intensity, endurance exercise 117. In any case, it is unknown yet whether exercise might affect the mechanisms of cellular senescence in an anti- or pro-melanoma way 118.

## Clinical implications of physical exercise in melanoma patients

Epidemiological evidence of health beneficial effects of exercise promoted the application of training protocols as an adjunct therapy for adult patients with chronic diseases, including cancer 119. However, poor evidence is currently available concerning feasibility and benefits of physical activity in melanoma patients. Moreover, only few clinical studies have been conducted to examine the feasibility of exercise protocols executed during cancer therapy 120.

Physical exercise was reported to induce immunological alterations in the tumor microenvironment of preclinical cancer models, largely mediated by the mobilization and redistribution of immune cells, thus suggesting a potential synergistic effect with immunotherapy 121. In the case of melanoma, mobilization and redistribution of both T and NK cells to the tumor were described as the putative mechanisms allowing exercise to decrease tumor incidence and progression towards metastasis 68. However, preclinical studies in melanoma murine models, aiming at showing the desirable synergism with ICIs, were unsuccessful. Nonetheless, a recent clinical study tried to define feasibility, safety, and effects of a telehealth exercise program in patients (n = 11) with advanced melanoma (stage III-IV) receiving ICIs 122. The 8-weeks long intervention included resistance, aerobic, and balance exercises, undertaken three times per week, with assessments at baseline and post-intervention. A statistically significant improvement in terms of cardiovascular capacity, static balance, upper body strength/endurance, was observed; moreover, no adverse events were reported. These outcomes led to conclude that the telehealth exercise intervention was feasible, safe, and tolerable for patients with melanoma receiving ICIs, which notoriously cause a reduction in patients' quality of life 122.

Previously, another completed clinical trial (i-Move - ACTRN12619000952145) was conducted to assess safety and acceptability of an individualized, 12-week, semi-supervised exercise program among patients (n = 30) with advanced melanoma (stage IV) scheduled to start immunotherapy (ipilimumab, nivolumab or pembrolizumab as single agents, or ipilimumab plus nivolumab). Secondarily, the trial aimed at providing preliminary evidence of efficacy of the exercise program (including moderate intensity aerobic exercise, resistance training exercises, and stretching of the major muscle groups) in counteracting immunotherapy-related fatigue and other patient-reported outcomes, like health-related quality of life, cancer symptoms, and psychosocial endpoints 123. However, to date, no published results are available.

The same approach is followed in a still active, not recruiting, phase 2/3 clinical trial (QUALIOR - NCT03169075) enrolling patients (phase 2, n = 120; phase 3, n = 312) with metastatic cancers, including melanoma, and receiving oral targeted therapy (i.e., tyrosine kinase, epidermal growth factor receptor [EGFR], cyclin-dependent kinases, BRAF, anaplastic lymphoma kinase [ALK], mTOR, and poly (ADP-ribose) polymerase [PARP] inhibitors). Being fatigue a frequent side effect following this treatment, reducing patient's adherence to the therapy and quality of life, a supervised home-based physical exercise program is adopted and assessed in terms of feasibility and physical benefit. Duration of the exercise program is 3 months, with a total of 12 supervised sessions (one weekly, aimed at reinforcing the musculoskeletal and cardiorespiratory system) and 24 non-supervised sessions (twice weekly, 30/60 minutes of walk, running, or biking, according to the patient's level of fatigue) 124.

In order to obtain a comprehensive view of the current status of clinical studies involving physical exercise in melanoma treatment, a search on the registry of clinical trials - ClinicalTrials.gov (available at https://clinicaltrials.gov) - was performed (September 18th, 2023). By using keywords like “melanoma” as condition or disease and “physical activity” or “exercise” as intervention/treatment, a total of 135 and 14 results were retrieved, respectively. Further in-depth analysis allowed the identification of 6 clinical trials properly addressing the clinical application of exercise regimens on melanoma patients. Details concerning these clinical trials, for whom no published results are available, are reported in **Table 1**.

## Discussion

Nowadays, the impact of physical activity/exercise on melanoma hallmarks results to be mostly investigated in preclinical *in vivo* studies, mainly performed by using female mice injected with syngeneic B16 melanoma cells and undergoing voluntary or non-voluntary, moderate- or high- intensity exercise on a treadmill. Although not supported yet by a consistent amount of clinical literature, the available data clearly point towards a modulating effect of training that hinders melanoma onset and progression. Among melanoma hallmarks, processes like metastasis formation, immune escape, and cancer-associated inflammation appear to be hampered by physical activity/exercise. Concomitantly, processes like neoangiogenesis, energy metabolism, and cancer cell death may be controlled in a direction promoting the health status of the individual when malignancy and training coexist.

The reported data are consistent with the results of a recent systematic review, based on PubMed, Web of Science, EMBASE, CINAHL, and SPORTDiscus databases, which reported the current evidence on the effects of training in patients with cutaneous melanoma, evaluated by objectively measured outcomes (cardiorespiratory fitness, physical function, body composition) and patient-referred indications about fatigue, cognitive function, psychological distress, treatment-related side effects. A total of six studies (2 retrospective analyses, 2 cross-sectional surveys, 2 non-randomized intervention trials) 125-130 and 882 melanoma patients were included in the analysis, ranging from stage I and/or II (localized), stage III (regional), and stage IV (metastatic) melanoma. A positive association between physical activity and quality of life maintenance during or after cancer treatment in melanoma survivors was reported, without data supporting an adverse impact 120. However, whether pre-diagnosis exercise might correlate with the prognosis of invasive melanoma is still unclear 131. Moreover, it seems that post-diagnosis exercise is not always preserved. Indeed, a decrease in leisure physical activity after treatment of early-stage melanoma was reported, in particular at 12 months after surgery, with a positive correlation with older age and female sex 132.

Another recent systematic review and meta-analysis examined the association between physical activity or cardiorespiratory fitness and the risk of cutaneous melanoma, after adjusting for sun exposure and sensitivity 133. A total of 3 cohort studies 30,134,135 and 5 case-control studies 28,87,136-138 on physical activity and melanoma risk, and 1 cohort study 139 on cardiorespiratory fitness and melanoma risk were included. The main finding was the existence of a statistically significant difference between melanoma risk estimated for physical activity from cohort studies and case-control studies. Cohort studies indicated a statistically significant 27% increased risk of melanoma in physically active participants versus those inactive, whereas case-control studies indicated a statistically non-significant 15% reduction for the same comparison. In the only cohort study that examined the association between cardiorespiratory fitness and melanoma risk, a positive but statistically non-significant association was found. Thus, although a potential preventive role of physical activity and cardiorespiratory fitness against melanoma is biologically possible, it may be difficult for epidemiologic studies to detect such protective effect without a comprehensive adjustment for UV radiation-related skin damage.

Despite the scarcity of related literature, the combination of physical activity/exercise with anticancer pharmacological treatments seems to be worthy of interest 140. However, no promising preclinical evidence is presently available regarding the association with ICIs, the current standard of care of metastatic melanoma. Indeed, as discussed above, the combination of physical activity with anti-PD-1 or anti-PD-L1 mAbs did not further inhibit tumor growth, compared to ICIs or exercise alone 71,79.

A recent *in vivo* exploratory analysis of selected mitochondrial markers, from the skeletal muscle of female C57BL/6 mice with melanoma (B16F10 cell line) or breast cancer (EO771 cell line) 141, suggested that exposure to both isotype control (IgG2a) and anti-PD-1 antibodies can modify the response to exercise of skeletal muscle mitochondria in a tumor-type dependent mode. In mice with B16F10 tumors, anti-PD-1-treatment combined with wheels running increased the expression of most measured markers associated with the activity of skeletal muscle mitochondria, while exposure to IgG2a did not increase the skeletal muscle mitochondrial content after training. In mice with EO771 breast cancer, both IgG2a and anti-PD-1 significantly increased the levels of these markers. Thus, in mice with melanoma, while IgG2a administration prevents the skeletal muscle mitochondria adaptation to exercise, the anti-PD-1 therapy is able to preserve the typical increase in mitochondrial protein content occurring with training. These data can pave the way for an exciting scenario, advocating exercise efficacy to prevent the muscle weakness and atrophy, typically occurring in animal models and patients undergoing immunotherapy 142-144.

In the context of neoangiogenesis cancer hallmark, an aerobic moderate intensity treadmill running, performed by C57BL/6J mice carrying B16F10 melanoma cells, significantly enhanced the antitumor effect of doxorubicin, likely as a result of vascular normalization induced by aerobic exercise 56. Doxorubicin is an anthracycline antineoplastic agent widely used against a range of solid tumors, like carcinomas and sarcomas, as well as hematological malignancies, but it is not approved for melanoma. Current research is aimed at finding innovative drug delivery systems with reduced systemic toxicity, to render doxorubicin also available for melanoma 145,146. Moreover, the clinical use of doxorubicin is limited by a progressive and dose-dependent, early or late-onset, cardiotoxicity, the latter occurring even up to 15 years after anthracycline administration 147. In this regard, a study published almost 10 years ago reported that, even at low intensity, exercise can promote tumor response to doxorubicin in male C57BL/6 mice injected with B16F10 melanoma cells, but it did not attenuate the cardiac fibrosis caused by the drug 148.

Another interesting scenario, where physical exercise seems to potentiate an experimental approach against melanoma is based on ceramide-induced cell death. Among sphingolipids, ceramide is one of the most bioactive molecules, with a key role in cell proliferation, death, and invasion 149, mainly by promoting apoptosis of tumor cells. The metabolism of sphingolipids is altered in cutaneous melanoma, favoring the accumulation of tumor promoting metabolites 150-151: dysregulated metabolic enzymes limit the accumulation of ceramide, also referred to as an anti-oncometabolite, and, conversely, promote the production of the tumor-promoting sphingosine 1-phosphate (S1P). Therefore, the balance between ceramide and S1P determines whether a cell undergoes death or proliferation. However, there is not yet a clinically relevant method to increase intracellular ceramide levels, as a potential anticancer strategy for melanoma with dysregulated ceramide metabolism, although exogenous ceramide analogues, as well as anti-S1P approaches, have been tested in several preclinical studies 152. Interestingly, moderate aerobic physical exercise, conducted in melanoma carrying male C57BL/6J mice, was reported to increase ceramide levels and activate the pro-apoptotic p53 signaling pathway, hence highlighting its efficacy as adjuvant therapy by modulating sphingolipid metabolism toward a pro-apoptotic ceramide signaling, at the expense of S1P activity 60. Moreover, it has been reported that the increase of the ceramide/S1P ratio can impair the expression of specific G protein-coupled receptors (S1P receptors, S1PRs) on which S1P acts as a ligand and, more interestingly, can exert a powerful antitumor activity in melanoma cells showing resistance to BRAFi 153.

Finally, the evidence that IL-15 up-modulation might, at least in part, explain the anticancer potential of physical activity 88, raises interesting cues on the option to combine exercise with strategies targeting such interleukin. Indeed, IL-15 is a pluripotent member of the immunoregulatory cytokines family, crucial in the generation and maintenance of NK cells and CD8+ memory T cells. Cytokine therapy is now emerging as a promising strategy to boost the host antitumor immune response, and recombinant forms of human IL-15 have been tested in clinical trials, also in patients with metastatic malignancies. However, although recognized as an immunotherapeutic candidate, the stability and bioavailability of recombinant IL-15 represent a drawback, and different forms of toxicity and adverse effects have been reported 154. One study proposed electroporation to deliver a plasmid carrying human *IL-15* gene directly to B16F10 melanoma nodules induced in female C57BL/6J mice, and this strategy resulted in regression of the tumor, long-term survival, lower tumor recurrence 155. Despite more work has to be done for developing a feasible IL-15-based therapy against cancer 156-157, the demonstrated interplay with physical activity should encourage further studies within this area of anticancer research.

**Figure 3:**
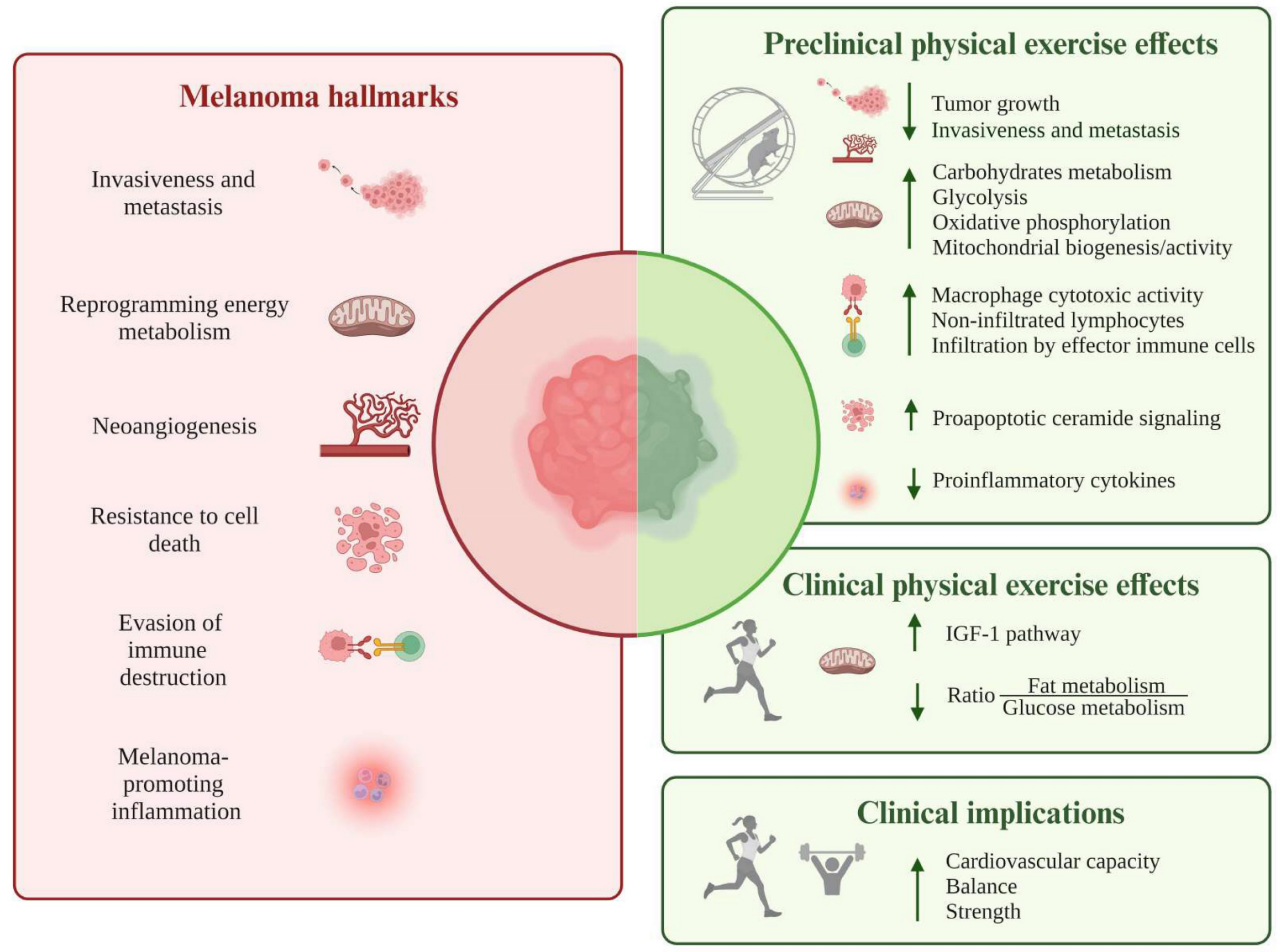
Schematic illustration of the available experimental results addressing how physical activity/exercise could modulate the following melanoma hallmarks: invasion and metastasis, reprogramming of energy metabolism, angiogenesis, resistance to cell death, evasion from immune destruction, and tumor-promoting inflammation. Most data originate from preclinical studies in murine melanoma models, pointing towards a decrease in tumor growth, metastatic behavior, and release of pro-inflammatory cytokines. Potentiation of antitumor immunity, promotion of pro-apoptotic pathways, and reprogramming of metabolic processes represent other biological mechanisms through which exercise may hamper melanoma evolution. The only available clinical data, produced by a prospective study on human healthy volunteers, suggest the reprogramming of energy metabolism, promoted by regular physical activity, as a protective factor against the development of metastatic tumors, including melanoma. Abbreviations. IGF-1: insulin-like growth factor-1.

Overall, the present review, for the first time, summarizes the available experimental evidence on how physical activity/exercise could modulate melanoma hallmarks (see **Figure 3** and **Table S1**), potentially affecting its onset, progression, and recurrence. Despite the involved signaling pathways and molecules have been mainly identified in preclinical studies in murine melanoma models, such evidence could represent the starting point for a deeper characterization, at the molecular level, of melanoma patients' response to regular training, thus enriching the currently available clinical literature, mainly represented by population-based cohort studies 32,33,131. Moreover, it might be interesting to focus attention on the distinctive hallmarks of melanoma around which there is limited or missing debate. Indeed, although the pathways by which exercise can attenuate carcinogenesis were described for hallmarks like sustaining proliferative signaling, evading growth suppressors, enabling replicative immortality, unlocking phenotypic plasticity, nonmutational epigenetic reprogramming, the evidence regarding melanoma is still limited or even non-existent.

## Supplementary Material

Supplementary figure and table.

## Figures and Tables

**Table 1:** Clinical trials involving melanoma patients and physical activity as an interventional strategy.

ClinicalTrials.gov identifier code	Title	Status, Country	Type of exercise intervention	Study plan	Endpoints
NCT05615883	Effects of acute and chronic exercise on myeloid-derived suppressor cells in melanoma patients	Recruiting, Italy	Acute exercise program: 80 minutes single walking session on a treadmill; chronic exercise program: 80 minutes walking session on a treadmill, repeated 3 times/week for 3 weeks.	Pre/post intervention, two-arms, sequential study; eligibility for patients with newly diagnosed melanoma.	To verify whether short-term physical exercise is a feasible and safe tool for re-setting antitumor immunity in early melanoma patients, by investigating how exercise modifies systemic immunity or biochemical/metabolic parameters.
NCT04866810	The Effect of Diet and Exercise on ImmuNotherapy and the Microbiome	Recruiting, United States	At least 150 minutes of moderate or 75 minutes of high-intensity exercise per week.	Intervention arm receiving high fiber, plant-based diet plus exercise prescription; control arm receiving standard diet and exercise recommendations. Eligibility for patients with confirmed melanoma not treated with any systemic therapy in the past 30 days and planning to undergo immunotherapy.	Feasibility of conducting a decentralized clinical trial involving diet and exercise prescriptions in melanoma patients receiving immunotherapy.
NCT06008977	Exercise to Boost Response to Checkpoint Blockade Immunotherapy	Not yet recruiting, United States	Supervised pedaling on an ergometer (stationary bike) at a moderate pace for a goal of 30 minutes.	Ten patients, receiving adjuvant or neoadjuvant immunotherapy, will be randomized to the exercise intervention arm and 10 to the no exercise arm. Eligibility: diagnosis of cutaneous melanoma for the adjuvant setting; cutaneous melanoma, cutaneous squamous cell cancer, or Merkel cell carcinoma for the neoadjuvant setting.	Relapse-free survival (adjuvant setting); pathological complete response (neoadjuvant setting).
NCT03171064	Exercise as a Supportive Measure for Patients Undergoing Checkpoint-inhibitor Treatment	Completed, Germany	Machine-based, 2 times/week endurance and resistance training, for a total of 12 weeks.	Intervention arm performing resistance and endurance exercise; control group receiving usual care. Eligibility: diagnosis of melanoma (independent of stage) and assignment of immunotherapeutic regimen.	Feasibility of the exercise intervention, quality of life, fatigue, sleep quality, depression, physical activity behavior, cardiopulmonary fitness, muscle strength, pain.
NCT0382591	3-month Aerobic and Resistance Exercise Intervention for Individuals Diagnosed With Melanoma	Terminated, United States	Session 1: aerobic exercise (20-30 minutes) followed by resistance training (10 stacked-weight machines, for 1-2 sets of 8-12 repetitions). Session 2: resistance bands and body weight exercises (4-6 exercises, 2 sets of 10-20 repetitions) followed by core exercises (2-3 sets of 10-20 repetitions) and aerobic training (20-30 minutes) at the end.	Active comparator: exercise intervention; sham comparator: wellness intervention (10 lectures in nutrition, exercise, and other integrative medicine topics, implemented among non-cancer individuals). Eligibility: melanoma patients within one year from diagnosis.	Feasibility and adherence, quality of life, cardiorespiratory fitness, muscle strength, functional status, nutritional intake, sun exposure, fatigue.
NCT05827289	Cancer Patients Better Life Experience	Recruiting, Netherlands	Mindfulness or yoga.	Single arm receiving the device Cancer Patients Better Life Experience (CAPABLE), aimed at symptom monitoring, information needs fulfilment and interventions, to improve mental- and physical wellbeing available as a smartphone application. Eligibility: confirmed stage III/IV melanoma receiving ICIs.	Change in fatigue, health-related quality of life, physical symptoms, melanoma specific quality of life, immunotherapy-related toxicity, psychological distress, satisfaction, recruitment rate, patient compliance, usability.

## Abbreviations

ALK: anaplastic lymphoma kinase
BRAFi: BRAF inhibitor
BT: body temperature
CerS6: ceramide synthase 6
COX-2: cyclooxygenase 2
CTLA-4: cytotoxic T lymphocyte-associated protein 4
DEGs: differentially expressed genes
EGFR: epidermal growth factor receptor
EMA: European Medicines Agency
FDA: Food and Drug Administration
GLUTs: glucose transporters
ICIs: immune checkpoint inhibitors
IGF-1: insulin-like growth factor-1
IL-2: interleukin 2
IL-4: interleukin 4
IL-6: interleukin 6
IL-10: interleukin 10
IL-15: interleukin 15
IL-17: interleukin 17
IFNγ: interferon-γ
mAb: monoclonal antibody
MAPK/ERK: mitogen-activated protein kinases/extracellular signal-regulated kinase
MEK: mitogen-activated protein kinase kinase
MEKi: MEK inhibitor
MMPs: matrix metalloproteinases
NK: natural killer cells
NKT: natural killer T cells
NFAT: nuclear factor of activated T cells
PARP: poly (ADP-ribose) polymerase
PD-1: programmed cell death 1
PD-L1: programmed death-ligand 1
PI3K: phosphoinositide-3-kinase
S1P: sphingosine 1-phosphate
S1PRs: sphingosine 1-phosphate receptors
TNF-α: tumor necrosis factor α
TT: thermoneutral temperature
TSP-1: thrombospondin-1
UV: ultraviolet
VCAM-1: vascular cell adhesion molecule 1
VEGF-A: vascular endothelial growth factor-A
VEGFR-1: vascular endothelial growth factor receptor-1
