# Supplementary material for: Impact of Physical Exercise on Melanoma Hallmarks: Current Status of Preclinical and Clinical Research
Source: J Cancer. 2024 Jan 1;15(1):1–19. doi: 10.7150/jca.88559 (PMC10751671; doi:10.7150/jca.88559)
Supplement: Supplementary file 1 — Supplementary figure and table. [file jcav15p0001s1.pdf]

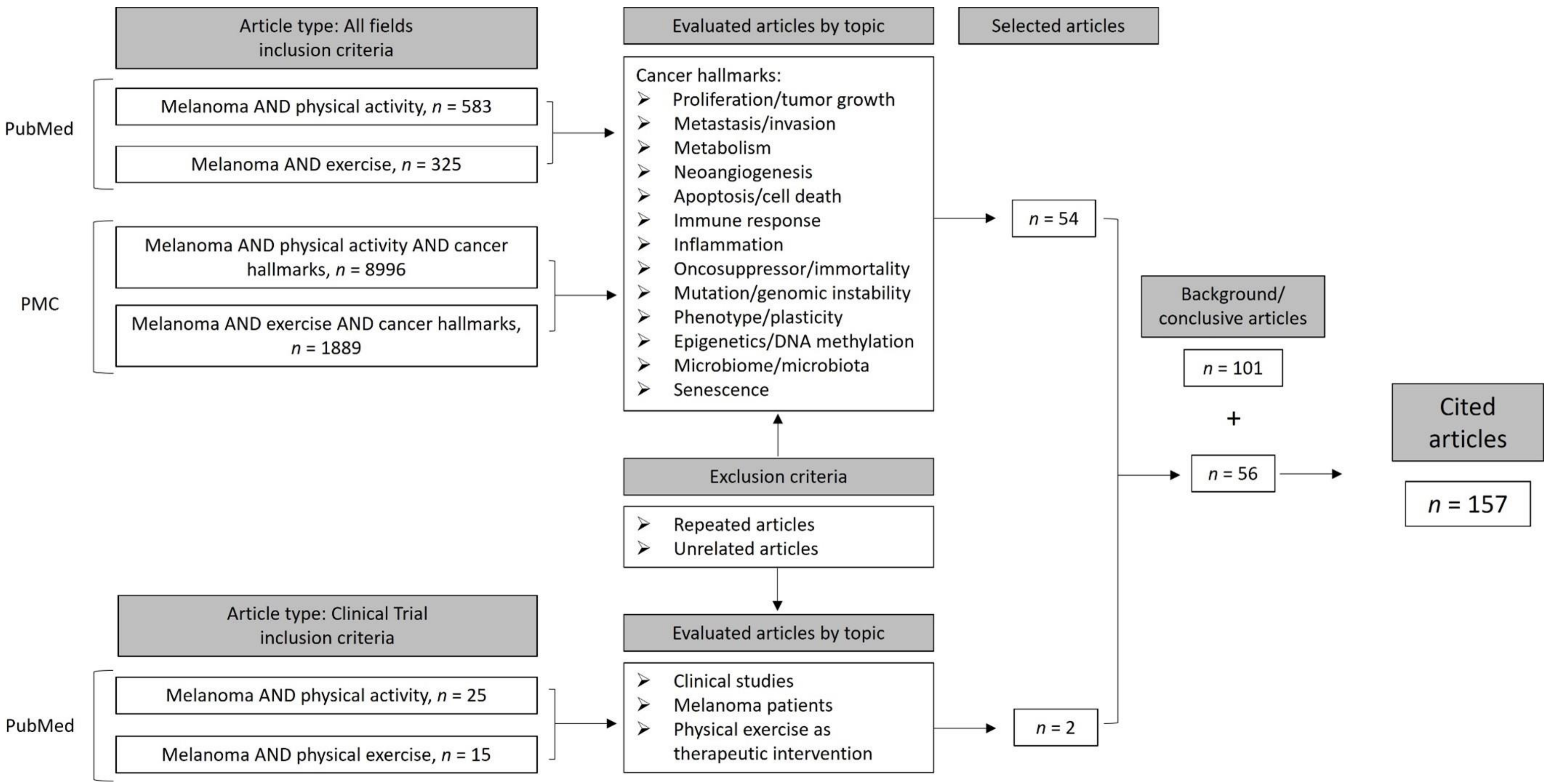

Figure S1. Flow chart of the bibliographic search strategy for selection of articles fully addressing the main topic of the present narrative review. A total of 583 and 325 articles were retrieved from PubMed (last access September 21st, 2023) by using the queries (melanoma) AND (physical

activity) or (melanoma) AND (exercise), respectively. Afterwards, 54 articles that matched the inclusion criteria (i.e., the indicated cancer hallmarks) and fitted with the indicated melanoma hallmarks were selected by authors' manual analysis.

Search in PubMed Central (PMC, last access September 21st, 2023) retrieved 8996 items by the use of (melanoma) AND (physical activity) AND (cancer hallmarks) queries, and 1899 items by the use of (melanoma) AND (exercise) AND (cancer hallmarks) queries. Following authors' manual selection, no articles from PMC were added to those retrieved from PubMed search.

Search on PubMed, by using "clinical trial" as article type and the queries (melanoma) and (physical activity) or (physical exercise), retrieved 25 and 15 results, respectively, but only 2 were relevant to the clinical applications of physical activity to patients with melanoma.

In all cases, exclusion criteria included repeated and unrelated articles. Other 101 papers were useful to provide background information to the results of the specific PubMed and PMC search, and to draw a critical conclusion, assessing whether a non-sedentary lifestyle might enhance the anti-melanoma potential of currently available or under investigation pharmacological strategies, for a total of 157 cited manuscripts.

Table S1. Experimental evidence linking physical activity to melanoma hallmarks.

Melanoma hallmark	Ref.	Type of study	Experimental model	Type of exercise intervention	Effects of physical exercise: Modulated molecules	*In vitro* evidence	*In vivo* evidence	Clinical evidence
**Invasiveness/ metastasis**	[39]	Preclinical *in vitro* and *in vivo* study	C57BL/6 mice carrying B16 melanoma cells.	Pre-tumor initiation treadmill running (30 minutes at 18 m/min) or running and fatigued (first run at 18 m/min, followed by increase of the speed (3 m/min every 30 min) until fatigue (~3 hours).	-	Reduced B16 melanoma cells growth in co-culture with alveolar macrophages from exercised mice, compared to co-culture with alveolar macrophages from resting animals.	Reduced formation of tumor foci in the lungs in exercised mice, compared to control resting animals.	-
	[40]	Preclinical *in vitro* and *in vivo* study	C57BL/6 mice carrying B16 melanoma cells.	Pre-tumor initiation short-term, moderate training (1 hour bout of treadmill running for 6 consecutive days), in combination with oat β-glucan consumption.	-	Increased antitumor cytotoxicity of macrophages from exercised mice, compared to unexercised mice.	Decreased metastatic spread of B16 melanoma cells in exercised mice, without additive effects with the intake of oat β-glucan.	-
	[42]	Preclinical *in vivo* study	C57BL/6 mice carrying B16BL/6 melanoma cells.	Voluntary running in freely accessible in-cage running wheels.	-	-	Absence of difference in number and size of lung metastases between sedentary and running mice.	-
	[43]	Preclinical *in vivo* and	Subdermal, intracarotid, or	Running on the treadmill before	-	-	Protection against metastasis to distant	Reduced incidence of

		clinical cohort study	intrasplenic injection of Ret-melanoma cell in C57BL/6 mice<br><br>Human cohort of healthy volunteers	tumor injection (20 minutes, 18 cm/s for 5 minutes, increasing the speed up to 24 cm/s for 8 minutes).<br><br>Routinely performed high-intensity exercise			organs.	invasive cancers (by 73%), compared to inactivity (follow-up of 20 years).
**Reprogramming of energy metabolism**	[43]	Preclinical *in vitro* and *in vivo*, and clinical cohort study	Subdermal, intracarotid, or intrasplenic injection of Ret-melanoma cell in C57BL/6 mice<br><br>Human cohort of healthy volunteers	Running on the treadmill before tumor injection (20 minutes, 18 cm/s for 5 minutes, increasing the speed up to 24 cm/s for 8 minutes).<br><br>High-intensity exercise for at least 24 hours prior to test.	Increased expression of Glut1, Glut2, and Glut4 mRNAs in organs from active mice, compared to inactive mice.<br><br>Upregulated expression of proteins belonging to the IGF-1 pathway.	Resumed tumor growth in the co-culture of lungs primary cells from active mice with B16 melanoma cells, after treatment with the mTor inhibitor rapamycin.	Protection against metastasis due to reprogramming of host organs metabolic habits (upregulation of carbohydrates metabolism, glycolysis, oxidative phosphorylation, and mitochondrial biogenesis/activity).	Shift in macronutrient utilization in association with exercise as a protecting factor against development of highly metastatic tumors.
**Neoangiogenesis**	[56]	Preclinical *in vivo* study	C57BL/6 mice carrying B16F10 melanoma cells	Post-tumor injection treadmill running (45 minutes, 5 days per week, at 10 m/min).	Activation of NFAT transcription factor.<br><br>Enhanced secretion of TSP-1.	-	Increased vessel length and blood supply; enhanced antiproliferative effects of chemotherapy (doxorubicin).	-
	[57]	Preclinical *in vivo* study	C57BL/6 mice carrying B16F10	Short-term post-tumor implantation physical activity on	-	-	Unaffected vascularity (unchanged CD31-	-

			melanoma cells	modified exercise wheels, allowing to count revolutions and quantify running distance.			positive vessel density), hypoxic state, and tumor growth rate, with respect to unexercised mice.	
	[58]	Preclinical *in vivo* study	C57Bl/6 mice carrying B16F10 and YUMMER 1.7 melanoma cells	Post-tumor implantation exercise, consisting of treadmill running for 45 minutes/day at 12 m/minute for 12/14 consecutive days.	Upregulated endothelial VCAM-1 expression.	-	Remodeling of melanoma-associated vasculature, and decreased vessel permeability, without change in tumor perfusion, compared to sedentary animals.	-
**Resistance to cell death**	[60]	Preclinical *in vivo* study	C57BL/6 mice bearing B16F10 or BP melanoma cells	Moderate intensity aerobic treadmill running (at 12 m/min for 45 minutes, 5 consecutive days/week, for a total of 2 weeks).	Upregulated canonical p53 signaling pathway.	-	Increased pro-apoptotic ceramide signaling and sensitivity to doxorubicin in mice bearing B16F10, but not BP, melanoma cells.	-
**Evasion of immune destruction**	[68]	Preclinical *in vivo* study	C57BL/6 mice carrying B16 melanoma cells	Voluntary wheel running, with access to running wheels 4 weeks before tumor cell inoculation and during tumor challenge; evaluation of total running distance by placement	Increased release of epinephrine.<br>Increased IL-6 plasma concentration.<br>Increased expression of mRNA coding for	-	Reduced tumor growth due to significantly increased infiltration by effector immune cells (T, NK, and dendritic cells).	-

				of bicycle computers on the running wheels.	ligands (H60a, Clr-b, MULT1), cytokines (IFNγ, IL-2, IL-15), and chemokines (CXCL10, CCL3, CX3CL1) involved in NK cell activation and chemotaxis.			
	[70]	Preclinical *in vivo* study	C57BL/6 mice carrying ovalbumin (OVA) expressing B16F10 melanoma cells	Voluntary wheel running monitored wirelessly by using an appropriate software.	-	-	Increased survival and decreased tumor growth rate in sedentary mice and receiving CD8+ T cells from exercising mice, compared to animals receiving T cells from non-exercising mice.	-
	[72]	Preclinical *in vivo* study	Chow- and high-fat fed C57BL/6 mice, carrying B16F10 melanoma cells	Voluntary wheel running before tumor cell inoculation and/or during tumor challenge, by monitoring of running distance.	Upregulation of markers of macrophages (CD68, CD74, and CD209), NK cells (NKG2D and NK1.1), and T cells (CD8, PDCD10, perforin, GrmM).	-	Suppressed tumor growth across chow and high-fat diets, although with an attenuated exercise-induced innate immune recognition of tumors in association with high-fat feeding.	-

	[58]	Preclinical *in vivo* study	C57Bl/6 mice carrying B16F10 and YUMMER 1.7 melanoma cells	Post-tumor implantation aerobic exercise, consisting of treadmill running for 45 minutes/day at 12 m/minute for 12/14 consecutive days.	-	-	Differentially regulated antitumor immune response and melanoma growth: significantly increased CD8+ T cell infiltration and reduced tumor size in YUMMER, but not in B16F10, murine melanoma models.<br>Unaffected antitumor activity of an anti-PD1 mAb in both melanoma preclinical models.	-
	[71]	Preclinical *in vivo* study	C57Bl/6NTac mice carrying B16 melanoma cells	Voluntary exercise on running wheels, with distance calculation, for 5 weeks prior to tumor cell inoculation.	Upregulated expression of PD-1, PD-L1 and PD-L2 immune checkpoints, CD28 co-stimulatory molecule, and CD28 ligands (B7.1 and B7.2).	-	Decreased melanoma growth, without statistically significant interaction with anti-PD-L1 or anti-PD-1 therapy.	-

	[79]	Preclinical *in vivo* study	C57BL/6 mice carrying B16F10 melanoma cells	Post-tumor implantation voluntary running on wheels equipped with magnetic sensor and digital counter to quantify revolutions.	-	-	Modified tumor immune microenvironment: reduced amount of CD8+ T cells within the total T CD3+ cell population (shift towards a more immunosuppressive phenotype), without changes in the amount of tumor infiltrating NK cells, between unexercised and exercised mice.	-
	[80]	Preclinical *in vitro* and *in vivo* study	C57BL/6 mice carrying B16F10 melanoma cells	Post-tumor implantation aquatic exercise in thermoneutral temperature (TT, 29°C) or body temperature (BT, 36°C) water, for 30 minutes, 6 days/week.	Enhanced release of IFNγ by CD8+ T cells from mice swimming in TT, compared to CD8+ T cells from mice exercised in BT.	Increased amount of splenocytes in mice swimming in TT, compared to mice exercised in BT, in particular CD8+ T, γδT, NKT, and NK cells.	Impaired tumor growth in mice swimming in TT, compared to mice exercised in BT.	-

**Melanoma-promoting inflammation**	[86]	Preclinical *in vivo* study	C57BL/6 mice carrying B16F10 melanoma cells	Moderate intensity treadmill exercise, for 1 h, 5 days/week, over 10 weeks, before tumor implantation.	Decreased serum levels of leptin and secretion of pro-inflammatory cytokines (IFN-γ, IL-2, and TNF-α), in mice on high-fat diet.	-	Reduced tumor growth in mice on a high-fat diet, and increased proliferation of tumor non-infiltrated lymphocytes, regardless of dietary regimen.	-

Abbreviations. BT: body temperature; GLUTs: glucose transporters; IFNγ: interferon-γ; IGF-1: insulin-like growth factor-1; IL-2: interleukin 2; IL-6: interleukin 6; IL-15: interleukin 15; NFAT: nuclear factor of activated T cells; NK: natural killer cells; NKT: natural killer T cells; PD-1: programmed cell death 1; PD-L1: programmed death-ligand 1; TNF-α: tumor necrosis factor α; TSP-1: thrombospondin-1; TT: thermoneutral temperature; VCAM-1: vascular cell adhesion molecule 1.
